# Hypoglycemia in patients with type 2 diabetes treated with oral antihyperglycemic agents detected by continuous glucose monitoring: a multi-center prospective observational study in Croatia

**DOI:** 10.1186/s12902-020-0518-5

**Published:** 2020-03-10

**Authors:** Maja Baretić, Valeria Bralić Lang

**Affiliations:** 1 Department of Endocrinology and Diabetes, University Hospital Centre Zagreb, Kišpatićeva 12, 10 000 Zagreb, Croatia; 2 School of Medicine, University of Zagreb, Šalata bb, Zagreb, Croatia; 3 Private Family Physician Office affiliated to University of Zagreb, School of Medicine, Zvonigradska 9, Zagreb, Croatia

**Keywords:** Type 2 diabetes, Continuous glucose monitoring, Hypoglycemia, Oral antihyperglycemic agents

## Abstract

**Background:**

Hypoglycemia in type 2 diabetes mellitus (T2DM) is still unsolved issue. The aim of this study was to investigate hypoglycemia in T2DM in participants treated with oral antihyperglycemic agents using different glucose cut-off values and to explore influence of different therapies.

**Methods:**

This multi-center prospective observational study included participant with T2DM from primary care offices across Croatia treated with antihyperglycemic agents who were monitored using professional continuous glucose monitoring (CGM) device (iPro™2). Hypoglycemia was defined as at least 1% of the monitored period spent in the hypoglycemic range and/or area under the curve of glycemia registered ever under the defined cut-off value. The higher upper limit of blood glucose cut-off value was 3.9 mmol/L (70 mg/dL) and the lower one 3.0 mmol/L (54 mg/dL).

**Results:**

Study included 94 participants. Median hemoglobin A1C levels, age, T2DM duration, body mass index, and CGM use duration were 7 (5.8–11.5) %, 65 (40–86) years, 7 (1–36) years, 30.4 (21.3–41.5) kg/m^2^ and 6 (1–7) days, respectively. Fifty participants were treated with sulfonylurea, primarily gliclazide (84%). The percentage of participant with hypoglycemia based on the higher cut-off value was 42.6% vs. 16% based on the higher cut-off value. The percentage of participant with nocturnal hypoglycemia (23 PM to 06 AM) was significantly lower among participant with hypoglycemia based on the higher cut-off value compared to lower one (7.8% vs. 22.9%). Sulfonylurea treatment did not influence the occurrence of hypoglycemia. Analysis of the data from participants having hypoglycemia based on the lower cut-off value pointed to other possible risk factors for hypoglycemia like prolonged overnight fasting, physical activity, alcohol consumption, and concomitant therapy with angiotensin-converting enzyme inhibitors.

**Conclusions:**

In participant with T2DM treated with oral antihyperglycemic agents hypoglycemia based on the blood glucose cut-off value of 3.9 mmol/L was more prevalent, but with less nocturnal hypoglycemia. Sulfonylurea therapy was not risk factor for hypoglycemia regardless of cut-off value. In participants having hypoglycemia based on the blood glucose cut-off value of 3.0 mmol/L some other possible factors were identified related to concomitant therapy, nutrition and daily habits.

**Trial registration:**

ClinicalTrials.gov Identifier: NCT03253237.

## Background

Continuous glucose monitoring (CGM) involves the use of an electrochemical enzymatic sensor to measure glucose in the interstitial fluid at regular intervals to provide valuable information in individuals with glycemic disturbances. The international consensus guidelines for utilizing, interpreting, and reporting CGM data state that self-monitoring of blood glucose (SMBG) is helpful in many patients, albeit with several limitations. In cases with discrepancies between the actual glycated hemoglobin (hemoglobin A1C) levels and values obtained using SMBG, CGM should be used. According to the International Consensus on Use of Continuous Glucose Monitoring statement, CGM data should be used to assess variations in hypoglycemia and glucose [1].

Professional CGM is performed by devices owned and managed by medical professionals; it uses blinded collection of glucose data. While wearing professional CGM devices, patients do not receive any information about glucose level deviations and do not change their daily habits; therefore, professional CGM devices observe true variations in glucose levels of the patients and provide valuable data on within-day and between-day variations in blood glucose as well as the frequency of unrecognized hypoglycemia. Food, activity, and therapy logs maintained by the patients are also helpful in the interpretation of glucose deviations. The advantages of CGM over SMBG for glycemic control in patients with type 1 diabetes mellitus are well established [2], whereas the advantages in patients with type 2 diabetes mellitus (T2DM) remain unclear. Numerous questions regarding therapeutic approaches in patients with T2DM remain unanswered; particularly those related to unrecognized hypoglycemia and postprandial glucose deviations. The Consensus Statement of the American Association of Clinical Endocrinologists and American College of Endocrinology has indicated that professional CGM should be considered in patients who have not reached their glycemic target after 3 months of the initial antihyperglycemic therapy and for those who require therapy that is associated with risks of hypoglycemia [3]. The summary also indicates that CGM is should be considered for those patients who are on intensive insulin therapy, for those with history of hypoglycemia unawareness, or those with recurrent hypoglycemia. The indications for CGM are evolving, with expansion of its implementation in more patients with T2DM [4].

Hypoglycemia remains a major barrier for tight glycemic control and a common complication of diabetes treatment. For patients with type 1 diabetes and T2DM, hypoglycemia remains one of the most enduring issues. Recent studies suggest that relevance of hypoglycemia in patients with T2DM is a misperception [5]. The rate of hypoglycemic events registered through SMBG in patients with T2DM ranges from 20 to 30%; hypoglycemic events in these patients are most commonly associated with insulin and/or sulfonylurea therapy [6–8]. Several studies have estimated even higher rates of hypoglycemia, up to 50%, in these patients [9]. Severe hypoglycemia occurs most frequently among patients with both extremely high and low upper and lower range of hemoglobin A1C values [10]. Studies using CGM have revealed that the percentage of hypoglycemic events is even higher than that previously appreciated, ranging from 57 to 79% in patients with T2DM on insulin therapy, with high percentage of nocturnal hypoglycemia [4, 11, 12]. Of note, different cut-off values for hypoglycemia have been used in different studies evaluating CGM in patients with T2DM [4, 11, 12]. The occurrence of hypoglycemia in patients with T2DM treated with insulin is well established. However, the prevalence of hypoglycemia in those treated with oral antihyperglycemic agents, who constitute the majority of patients with T2DM, remains unclear. According to the Joint Position Statement of the American Diabetes Association and the European Association for the Study of Diabetes, a blood glucose level of 3.9–3.0 mmol/L (70–54 mg/dL) is a hypoglycemia alert value and a blood glucose level of < 3.0 mmol/L (< 54 mg/dL) indicates serious, clinically important hypoglycemia. The International Hypoglycaemia Study Group recommends that the lower limit of the glucose cut-off levels for hypoglycemia is clinically significant and should be included in reports of clinical trials on glucose-lowering agents [13].

Given the significance of hypoglycemia in patients with T2DM and the limited knowledge of its frequency in patients on oral antihyperglycemic agents, we conducted this study aimed to reveal the occurrence of hypoglycemia in T2DM using different glucose cut-off values and the influence of different therapies in patients treated with oral antihyperglycemic agents. The hypothesis is that the occurrence of hypoglycemia in patients with T2DM treated with oral antihyperglycemic agents using glucose cut-off level of 3.9 mmol/L is high, but not mandatory associated with specific antidiabetic therapy.

## Methods

### Study design

This multi-center prospective observational study was conducted in 20 primary care offices. The study was registered at ClinicalTrials.gov with the identifier NCT03253237. It was approved by the Ethics Committee of the School of Medicine at University of Zagreb.

A total of 20 general practitioners from four Croatian regions recruited up to five participant with T2DM; all participants wore a CGM device (iPro™2; Medtronic Dublin, Ireland) for up to 7 days. Both the participants and physicians were blinded for CGM data until after the data were downloaded. The study period involved screening and two visits. Screening included collecting baseline patient history, particularly on diabetes and complications, and anthropometric and laboratory data and obtaining informed consent. At the first visit (day 1), the CGM device was initiated and participants were instructed to maintain a 7-day diary to include data on eating (time of day, what was eaten, alcohol consumption), physical exercise (type of activity, duration), drugs (type of medication, time of day, dose) and to perform SMBG four times a day. They were instructed to maintain usual daily habits related to physical activity, medication and meals. Participants were also educated regarding the symptoms of hypoglycemia and instructed to report hypoglycemia if observed using SMBG or based on the symptoms. A glucometer (Contour® Ascensia, Basel, Switzerland) was used for SMBG. At the second visit (day 7), the CGM device was removed.

Data were collected from the CGM device and uploaded to the CareLink iPro software. The following parameters were analyzed: glucose management indicator (GMI) calculated using the following formula: 12.71 + 4.70587 × (mean glucose); area under the curve (AUC); standard deviation (SD); percentage of time spent in specific blood glucose ranges (< 3.0, < 3.9, 3.9–10, and > 10 mmol/L); sensor-estimated average glucose levels and the percentage coefficient of variation for glucose levels (%CV) calculated using the following formula: ([SD of glucose levels]/[mean glucose levels] × 100).

### Study participants

A total of 100 participants who fulfilled the following criteria were included in the study: diagnosis of T2DM for at least 1 year prior to study entry; age ≥ 40 years; and no subcutaneous therapy for diabetes such as insulin or glucagon-like peptide-1 receptor agonists. Exclusion criteria were as follows: known coagulopathies, oral anticoagulant therapy, skin disease that interferes with CGM application, febrile illness, and patient’s inability to physically visit the general practitioner’s office or respond to questionnaires. The indication for CGM included a clinical suspicion of hypoglycemia (anamnestic data showing otherwise unexplained shakiness, dizziness, sweating, hunger, irritability, etc.) or a discrepancy between actual blood glucose levels and hemoglobin A1C levels (i.e., having normal glucose levels according to SMBG but high A1C levels or vice versa). In six participants, CGM revealed no data. In four of the participants, the sensor was not properly attached due to the failure of the adhesive used for the attachment of the sensor to the skin during the extreme hot summer days. Additionally, in two participants, no records were uploaded to the software due to unknown reasons.

### Statistical analyses

All statistical analyses were performed using SPSS ver. 25.0 (IBM SPSS inc. 2017). Normality of distribution in individual parameters was determined using Shapiro–Wilk test. Because there was a significant deviation from normal distribution, medians and interquartile ranges were also shown in descriptive parameters along with means and standard deviations. When the rule of homogeneity of variance showed a significant difference (Levene’s test), nonparametric statistical procedures was used.

One-way analysis of variance (ANOVA) was used to compare independent means of the three groups with different sets of variables. In cases where variances were not homogenous (Levene’s test), Kruskal–Wallis test, as a nonparametric substitute for ANOVA, was used with Scheffe’s post hoc test; Mann–Whitney U-test was used to analyze the differences between two independent groups in the parameters of glucovariability measured using the sensor. Logistic regression analysis was used to determine the independent predictors of hypoglycemia.

## Results

Data from 94 Caucasian participants (38 males, 56 females) were included in the final analysis. The median age, duration of diabetes, and BMI were 65 (40–86) years, 7 (1–36) years, and 30.4 (21.3–41.5) kg/m^2^, respectively. Participants wore CGM for a median of 6 (1–7) days. Baseline characteristics of the participants included in the study are presented in Table 1 and the sensor with 7-day diary data of the participants included in the study are presented in Table 2.

**Table 1:** Baseline characteristics of the participants

GROUPS (all participants Caucasian)	Group 1 *N* = 54 glucose > 3.9 mmol/L		Group 2 *N* = 25 glucose 3.9–3 mmol/L		Group 3 *N* = 15 glucose < 3.0 mmol/L		Pairwise comparison (*p* value)		
							Group 1 vs. Group 2	Group 1 vs. Group 3	Group 2 vs. Group 3
	Median	SD	Median	SD	Median	SD			
**Age (years)**	64.50	8.03	67.00	8.55	63.00	8.63	0.17	0.86	0.24
**BMI (kg/m2)**	30.43	40.51	28.88	4.98	29.80	2.90	0.25	0.30	0.47
**Waist circumference (cm)**	102.00	11.00	98.00	11.93	105.00	12.41	0.47	0.16	0.19
**Hip circumference (cm)**	110.00	11.42	106.00	12.12	104.00	6.18	0.07	0.05	0.38
**Systolic BP (mmHg)**	130.00	11.36	130.00	9.95	130.00	14.60	0.33	0.10	0.18
**Diastolic BP (mmHg)**	80.00	6.30	78.00	5.40	80.00	9.10	0.17	0.10	0.04
**Pulse (beats/min)**	72.00	8.36	70.00	6.36	72.00	7.35	0.18	0.39	0.32
**Gliclazide dosage (mg)**	15.00	34.46	45.00	36.00	60.00	47.93	0.22	0.12	0.32
**Diabetes duration (years)**	6.00	5.48	8.00	5.02	7.00	3.99	0.07	0.43	0.14
**A1C (%)**	7.00	1.13	6.90	0.83	7.10	0.95	0.22	0.40	0.17
**eGFR MDRD (mL/min/1.73 m2)**	82.91	22.60	78.85	19.16	83.28	22.35	0.37	0.41	0.31
**Fasting glucose (mmol/l)**	8.00	1.91	7.30	1.53	7.70	1.53	0.04	0.13	0.38

***A1C*** Laboratory estimated hemoglobin A1C, ***BP*** Blood pressure, ***BMI*** Body mass index, ***eGFR-MDRD value*** *estimated glomerular* filtration rate using the *MDRD equation*

**Table 2:** Data collected from the CGM device and 7-day diary of the participants

GROUPS (all participants Caucasian)	Group 1 N = 54 glucose > 3.9 mmol/L		Group 2 N = 25 glucose 3.9–3 mmol/L		Group 3 N = 15 glucose < 3.0 mmol/L		Pairwise comparison (*p* value)		
	Median	SD	Median	SD	Median	SD	Group 1 vs. Group 2	Group 1 vs. Group 3	Group 2 vs. Group 3
**Physical activity (minutes)**	30.00	28.02	30.00	36.26	60.00	31.67	0.05	0.02	0.33
**Sensor glucose (mmol/l)**	6.80	1.33	5.80	0.52	6.10	1.01	0.00	0.02	0.03
**GMI**	44.70	6.27	40.00	2.45	41.42	4.73	0.00	0.02	0.03
**CV%**	41.00	10.32	33.53	9.29	25.52	4.75	0.01	0.00	0.00
**SD**	1.80	0.59	1.60	0.47	2.40	0.38	0.01	0.00	0.00

***GMI*** Glucose management indicator, ***%CV*** Percentage coefficient of variation for glucose levels, ***SD*** standard deviation

The percentage of time in range 3.9–10 mmol/L correlated negatively with the hemoglobin A1C value (*r* = − 0.42), GMI value (*r* = − 0.71), SD of all sensor-estimated glucose level variations (*r* = − 0.64), and GMI (*r* = − 0.8). The %CV correlated positively with the time with blood glucose levels < 3.9 mmol/L (r = 0.478), but there was no correlation of %CV with time with blood glucose levels < 10 mmol/L, laboratory-measured hemoglobin A1C levels or GMI values.

Hypoglycemia was defined as at least 1% of the CGM time spent in the range under the defined cut-off value (< 3.9 mmol/L or < 3.0 mmol/L) and/or the AUC of blood glucose registered ever under the defined higher or lower cut-off values. Nocturnal hypoglycemia was defined as a blood glucose value of < 3.9 mmol/L obtained from CGM data between 23 PM and 06 AM.

A total of 42.6% of the entire study cohort was defined to have hypoglycemia based on blood glucose levels < 3.9 mmol/L: 26.6% of them had hypoglycemia defined as blood glucose levels within the range of 3.9–3 mmol/L and 16% had hypoglycemia defined as blood glucose levels < 3.0 mmol/L.

The participants were divided into three groups according to blood glucose cut-off levels: group 1 comprising participants without hypoglycemia with blood glucose levels > 3.9 mmol/L (*n* = 54), group 2 with verified hypoglycemia with blood glucose levels in the range of 3.9–3.0 mmol/L (*n* = 25), and group 3 with verified hypoglycemia < 3.0 mmol/L (*n* = 15). There were no significant differences in baseline laboratory data, also data including age, duration of diabetes, blood pressure, puls, waist and hip circumference, days wearing CGM device among the three groups. Furthermore, there was a significant difference in GMI (*p* < 0.001) between groups. Other analyzed sensor data with Kruskal-Wallis test (SD, CV%, time spent in specific glucose ranges, nocturnal hypoglycemia and AUC) were significantly different among all three groups.

Among all analyzed participants, 13.8% had nephropathy (defined by either a reduced kidney function with an eGFR MDRD - estimated glomerular filtration rate using the MDRD equation of < 60 mL/min/1.73 m^2^ or albuminuria, i.e., albumin/creatinine ratio ≥ 3), 9.6% had retinopathy (diagnosed based on a comprehensive dilated eye exam), and 22.3% had neuropathy (diagnosed based on a physical exam and/or clinical symptoms). Chi-square test showed no significant difference regarding the occurrence of comorbidities among the three groups.

Groups 2 and 3 were separately analyzed using Mann–Whitney test regarding the sensor-collected data; a significant difference was observed between the two groups in sensor glucose, GMI, SD, %CV, time spent in specific glucose ranges and nocturnal hypoglycemic events (Table 3). There was no difference in data collected form 7-day diaries (minutes of physical activities) (Table 2).

**Table 3:** Pairwise comparisons between two groups of participants with different hypoglycaemia cut-off value of the data collected from the CGM device

GROUPS (all participants Caucasian)	Group 2 N = 25 glucose 3.9–3 mmol/L		Group 3 N = 15 glucose < 3.0 mmol/L		Mann-Whitney test
Distribution of sensor glucose level (% of time)	Median	SD	Median	SD	*p* value
**> 10 mmol/L**	3.00	7.06	13.00	15.08	0.00
**3.9–10 mmol/L**	92.00	9.73	76.00	13.74	0.00
**< 3.9 mmol/L**	2.00	7.68	7.00	13.72	0.02
**< 3 mmol/L**	0.00	0.00	3.00	12.00	0.00
**Nocturnal hypo. < 3.9 mmol/L**	5.00	10.23	13.00	29.02	0.03
**Nocturnal hypo. < 3.0 mmol/L**	0.00	0.00	3.00	6.93	0.00
**AUC < 3.9**	0.01	0.07	0.06	0.20	0.00
**AUC < 3**	0.00	0.00	0.02	0.09	0.00

**Nocturnal hypo**.- nocturnal hypoglycemia obtained from CGM data between 23 PM and 06 AM; **AUC**- area under the curve;

More than half of participants (52.1%) were treated with an ACE inhibitor; chi-square test showed no significant difference among the three groups regarding the use of ACE inhibitors. One, two, three, and four oral antihyperglycemic agents were used in 33, 41, 23, and 3% of the participants, respectively. Among the 50 participants treated with insulin secretagogues, the majority were treated with gliclazide (84%), whereas the remaining participants were treated with glimepiride or repaglinide. The dosages of glimepiride and repaglinide were converted to the approximate dose for gliclazide to achieve minimal equivalent dosages. Consequently, 50% of the participants in group 1 were treated with insulin secretagogue; in groups 2 and 3, 60 and 55% of the participants were treated with insulin secretagogues, respectively. Kruskall–Wallis test showed no difference in the dosage of sulfonylurea among the three groups.

The analysis of data on hypoglycemia symptoms based on the 7-day patient diaries revealed small number of participants with self-reported hypoglycemia, with no hypoglycemia symptoms reported during the nighttime. In groups 1, 2, and 3, five (9%), four (16%), and two (13%) participants, respectively, reported symptoms of hypoglycemia. Chi-square test showed no significant difference in the median dosage of sulfonylurea among the three groups (χ^2^ = 0.797, df = 2; *p* = 0.671). Among five participants reporting hypoglycemia symptoms in 7-day diaries in group 1, two were treated with sulfonylurea (receiving 90 mg and 60 mg of gliclazide per day); among four participants in group 2, two were treated with sulfonylurea (receiving 90 mg and 60 mg of gliclazide per day); and among two patient in group 3, one was treated with sulfonylurea (receiving 120 mg of gliclazide per day).

Fifteen participants in group 3 were analyzed separately as well. Seven participants in group 3 received gliclazide, including 120 and 60 mg/day in two and five participants, respectively. In addition, one patient from group 3 was on repaglinide therapy. CGM data analysis revealed that all participants on insulin secretagogue therapy experienced more than one episode of hypoglycemia, especially during the night. Seven participants had hypoglycemia (< 3.0 mmol/L) while not on sulfonylurea therapy. CGM data analysis revealed that hypoglycemia < 3.0 mmol/L occurred once, primarily during the night. Of the abovementioned seven participants, three participants were on metformin monotherapy, two participants were receiving two antihyperglycemic agents, and two participants were receiving three hypoglycemic agents. All three participants treated with metformin only were also receiving ACE inhibitors for hypertension. The anamnestic data collected from the 7-day diaries in two participants having hypoglycemia < 3.0 mmol/L and being treated only with metformin revealed vigorous exercise in the evening before the hypoglycemic event; one patient reported alcohol consumption (distilled spirit). Furthermore, two participants in group 3 treated with two hypoglycemic agents (one, metformin with vildagliptin; another, metformin with pioglitazone) were also on ACE inhibitor therapy. Two participants from group 3 were receiving three oral antihyperglycemic agents. The one patient on metformin, pioglitazone, and vildagliptin reported prolonged fasting for 12 h in the 7-day diary, whereas the other patient on metformin, pioglitazone, and sitagliptin was also on ACE inhibitor therapy for hypertension.

Logistic regression analysis was performed to identify independent risk factors for hypoglycemia < 3.0 mmol/L. Independent variables were ACE inhibitor therapy, sulfonylurea therapy, dosage of sulfonylurea, nephropathy, A1C levels, eGFR-MDRD value, minutes of physical activities, reporting hypoglycemia in the 7-day diary, SD, CV%, GMI and percentage of time spent in specific ranges. Logistic regression analysis showed that participants without nephropathy have a 95% less chance for developing hypoglycemia. Also, participant with low CV%, an indicator of glucovariability, have 17% less chance for developing hypoglycemia.

## Discussion

In this study, we found that percentage of participant with hypoglycemia based on the higher cut-off value of 3.9 mmol/L (42.6%) is much higher compared to those with hypoglycemia based on the lower cut-off value of 3.0 mmol/L (16%). The nocturnal hypoglycemic events were 3x more frequent in participant with hypoglycemia based on the lower cut-off value. Those participants had more glucovariability, they spent more time in both hypoglycemia and hyperglycemia (glucose > 10 mmol/L). In the present study, few participants reported the symptoms of hypoglycemia (no one nocturnal hypoglycemia) in their 7-day diary. Some participants reporting hypoglycemia belonged to the group without confirmed hypoglycemia at all. Although studies examining CGM in patients with T2DM are limited, similar findings have been reported before; a study including 108 patients revealed that approximately half of the participants (*n* = 53) experienced at least one episode of hypoglycemia during 5 days of CGM, in agreement with our findings, and that only 21% of the participants experienced blood glucose levels < 2.8 mmol/L [14].

In this study there are no data to support long-term or short-term morbidity or mortality outcomes for hypoglycemic events with different glucose cut-off values. Some trials, like The Action to Control Cardiovascular Risk in Diabetes (ACCORD), illustrated the significance of a lower cut-off blood glucose value. ACCORD showed that hypoglycemia with glucose levels < 2.8 mmol/L in T2DM is associated with mortality [15]. The occurrence of hypoglycemia in nondiabetic individuals has been clarified by studies using CGM [16, 17]. One of these studies described that out-of-range glucose values are likely to be observed during CGM [16]; this study revealed that almost none of the otherwise normoglycemic individuals experienced hypoglycemia with glucose levels < 3.3 mmoL/L, but hypoglycemia with levels < 3.9 mmol/L occurred during the day and night in 1.1 and 2.2% of the time, respectively. In another study including healthy individuals, blood glucose levels of ≤3.9 and ≤ 2.8 mmol/L were detected in 41 and 5.5% of the cohort, respectively [17]. Such CGM studies in healthy individuals facilitate the understanding of physiology of blood glucose regulation and reveal a lower “normal” glycemic range. Furthermore, these studies can prevent misinterpretation of hypoglycemia in patients with T2DM. Thus, had we included healthy participants without diabetes in the study, they would have been recognized as being hypoglycemic, not healthy.

Second interesting pattern disclosed in our study is that treatment with insulin secretagogues did not influence the occurrence of hypoglycemia. The percentage of participant on secretagogues was comparable among the groups of participant with or without verified hypoglycemia; i.e. half of the participants in the hypoglycemia group with a cut-off value of < 3.0 mmol/L were not receiving insulin secretagogue therapy at all. Moreover, some of the participants with self reported hypoglycemia were not treated with insulin secretagogue. Similar observation has been reported in a previously mentioned larger study on CGM in participant with T2DM, confirming that 18.9% of participant with hypoglycemic episodes were not using medications that typically cause hypoglycemia [14]. One potential explanation for this finding is the high rate of treatment with gliclazide used in our study, a sulfonylurea associated with the lowest risk of hypoglycemia among the sulfonylurea group of antidiabetics [18], although there are reports of hypoglycemia associated with gliclazide and glimepiride treatment [19]. One study reported a similar finding regarding CGM, hypoglycemia, and sulfonylurea in elderly diabetics; almost 21% of the study cohort had glucose levels < 3.0 mmol/L persisting for at least 15 min with or without symptoms [20]. The present study findings confirm the results of a systematic literature review of randomized clinical trials that revealed hypoglycemia in participant with T2DM while fasting during Ramadan. Those treated with either gliclazide or dipeptidyl peptidase-4 inhibitors had a comparable low risk of symptomatic hypoglycemia [21].

Another interesting issue is the possibility that some other unrecognized factors beyond sulfonylurea can cause hypoglycemia. Among the 15 participants in group with a cut-off value for hypoglycemia of < 3.0 mmol/L, 7 participants were not receiving insulin secretagogue therapy. We identified other possible risk factors including overnight prolonged fasting (> 8 h), physical activity, alcohol consumption, and concomitant therapy (i.e., ACE inhibitors for hypertension). Prolonged fasting is a known cause of hypoglycemia because blood glucose levels begin to drop several hours after a meal. In most cases, this condition occurs during the night. As previously mentioned, in CGM studies hypoglycemia occurs more often during the night in healthy individuals as well [16]. In our study, the proportion of participants with nocturnal hypoglycemia with lower cut off value was higher than that of those with higher cut off value for hypoglycemia within the range of 3.9–3.0 mmol/L (22.9% vs. 7.8%). Ethanol lowers the overnight secretion of growth hormone in nondiabetic subjects and reduces the response to hypoglycemia [22]. The effect of ethanol on nocturnal growth hormone levels in participant with diabetes is unknown and may be relevant to delayed hypoglycemia. Another possible factor underlying hypoglycemia is concomitant therapy with ACE inhibitors. The use of ACE inhibitors has been associated with increased insulin sensitivity in diabetic patients, and their concomitant use with antidiabetic therapies may facilitate their blood glucose-lowering effect with a concomitant risk of hypoglycemia [23]. Logistic regression analysis in this study showed that participants without nephropathy have a 95% less chance for developing hypoglycemia. Since median eGFR-MDRD was far above 60 mL/min in all three groups, diagnosis of nephropathy was, in majority of cases, a consequence of increased albuminuria. Such participant had a mandatory ACE inhibitor in their therapy.

Data collected from the 7-day diary entries highlight the importance of physical exercise in the occurrence of hypoglycemia too. In our study, minutes of physical activities were significantly associated with the occurrence of hypoglycemia, and they were higher in groups with lower cut off values for hypoglycemia. During prolonged exercise, the contribution of muscle glycogen declines physiologically and that of blood glucose increases to maintain the total carbohydrate oxidation rate required for exercise [24]. Insulin sensitivity was shown to increase with physical activity, allowing better usage of insulin in participant with T2DM [25]. In such situations, blood glucose levels begin to decline even in healthy individuals; those exercising for a longer period in a fasted state become hypoglycemic, with blood glucose levels < 3.0 mmol/L [2].

Finally, the interpretation of the sensor data is an important aspect of studies using CGM. The interpretation of professional CGM reports should review patterns of hypoglycemia, hyperglycemia, and measures of glucose variations in addition to hemoglobin A1C levels and time spent in a specific range. SD measures blood glucose directly, including the variations; it is a quite robust measure describing variations or dispersions of average. CGM studies in participant without diabetes have revealed that the normal range of SD is 0.0–3 mmol/L [25]. In the present study, the SD of the whole cohort was 1.9, indicating low variability in blood glucose. SD was significantly different among all three groups; it was highest in participants with hypoglycemia defined as blood glucose levels < 3.0 mmol/L confirming connection among hypoglycemia and glucovaribility. We found that larger swings of glucose predicted worse glucose control. The hypoglycemic swings appear to be more important because the % CV, also a valuable measure of glucose variations, correlated with the time spent in hypoglycemia but not with the time spent in hyperglycemia. Furthermore, logistic regression analysis confirmed that together with nephropathy, %CV predicts the outcome for hypoglycemia.

The major limitation of this study was the small sample size, limiting the statistical power of the study and hindering the generalization of the findings to other populations, together with the observational nature of the study as well as the short duration (7 days).

Additionally, the findings are applicable only to participant with T2DM on oral antihyperglycemic agents. Another limitation of this study is that all participants included were Caucasian and thus, this study data is not generalizable to other populations. Therefore, future investigations should include larger cohorts with different treatment approaches.

## Conclusions

We found that in a cohort of participant with T2DM treated with oral antihyperglycemic agents hypoglycemia based on the blood glucose cut-off value of 3.9 mmol/L was more prevalent than the one based on the blood glucose cut-off value of 3.0 mmol/L. Although the study participants exhibited low variability in blood glucose levels, the hypoglycemic swings appear to be more important. The nocturnal hypoglycemic events were more frequent in participant with hypoglycemia based on the lower cut-off value and they had more glucovariability. Insulin secretagogue therapy was not a risk factor for hypoglycemia regardless of cut-off value. However, some other possible risk factors for hypoglycemia were recognized, including prolonged overnight fasting, physical activity, alcohol consumption, and concomitant therapy with ACE inhibitors.

## Data Availability

The datasets used and/or analyzed during the current study available from the corresponding author on reasonable request.

## Abbreviations

%CV: the percentage coefficient of variation for glucose levels
ACCORD: Action to Control Cardiovascular Risk in Diabetes
ACE: Angiotensin-converting enzyme
AUC: Area under the curve
BMI: Body mass index
CGM: Continuous glucose monitoring
GMI: Glucose management
GMI: Glucose management indicator
SD: Standard deviation
SMBG: Self-monitoring of blood glucose
T2DM: Type 2 diabetes mellitus
